# Endovascular management of tandem embolic stroke due to cardioembolic free-floating thrombus: a case report

**DOI:** 10.3389/fnins.2025.1654601

**Published:** 2025-10-15

**Authors:** Bin Hu, Jing Li, Liang Li

**Affiliations:** Department of Neurology, Huangpi District People’s Hospital of Jianghan University, Wuhan, China

**Keywords:** embolic tandem lesion, free-floating thrombus, mechanical thrombectomy, stroke, case report

## Abstract

**Background:**

Tandem lesions (TLs), defined as simultaneous occlusions of both extracranial and intracranial arteries, represent a particularly challenging subset of large vessel occlusion (LVO) strokes. While most TLs are attributed to atherosclerotic changes or arterial dissection, a smaller subset originates from cardioembolic emboli arising from the heart or aortic arch, commonly referred to as embolic tandem lesions. In rare instances, these emboli form a free-floating thrombus (FFT) in the carotid artery, characterized by mobile intraluminal thrombotic material with high embolic potential. Optimal treatment strategies for these rare and complex cases remain undefined.

**Case summary:**

We present a rare case involving an embolic tandem lesion in a 73-year-old male with known atrial fibrillation who presented with acute ischemic stroke upon awakening. Imaging and angiography demonstrated a free-floating thrombus (FFT) at the brachiocephalic bifurcation extending into the origins of the right common carotid and right subclavian arteries, a free-floating thrombus at the carotid bifurcation, and an additional thrombus occluding the right ICA terminus and the origin of the M1 segment. A staged endovascular approach was employed: intracranial recanalization was first achieved via mechanical thrombectomy, followed by stent-assisted stabilization of the residual free-floating thrombus within the common carotid artery. The patient experienced significant neurological improvement and attained functional independence at 3-month follow-up.

**Conclusion:**

This case underscores the diagnostic and therapeutic complexity associated with embolic tandem lesions and carotid FFT involvement. Comprehensive preprocedural imaging, including assessment of the aortic arch and cervical vessels, is critical. A personalized, multi-modality endovascular approach combining thrombectomy and stenting can enable successful revascularization and favorable clinical outcomes, particularly when FFT complicates tandem occlusions.

## 1 Background

Acute ischemic stroke (AIS) remains a leading cause of neurological disability and death worldwide. The advent of endovascular therapy, particularly mechanical thrombectomy, has substantially improved reperfusion rates and clinical outcomes for strokes caused by large vessel occlusion (LVO) (Sharma and Lee, 2025). Among these cases, approximately 17% involve tandem lesions (TLs) defined by the simultaneous occlusion of an extracranial cervical artery and a distal intracranial vessel, typically in the anterior circulation (Assis et al., 2018). Due to their complex anatomy and variability in therapeutic approaches, TLs are widely considered among the most difficult LVO subtypes to treat via endovascular means.

The majority of proximal TLs arise from intrinsic vascular pathology such as atherosclerotic plaque rupture or arterial dissection, resulting in stenosis or occlusion. However, a subset of TLs stems from emboli that originate in the heart or aortic arch, often described as “embolic tandem lesions” (Papanagiotou et al., 2018). In particularly rare cases, a free-floating thrombus (FFT) forms in the carotid artery. This entity, known as a carotid free-floating thrombus (CFFT), typically consists of a thrombus partially anchored to the vessel wall, with its distal end freely oscillating within the lumen. This mobile configuration renders it highly embolic, with a heightened risk of distal migration during manipulation. Proposed mechanisms for CFFT include rupture of an atherosclerotic plaque, partial arrest of an embolus in transit, direct embolization from cardiac or aortic sources, arterial dissection, and prothrombotic conditions (Dowlatshahi et al., 2023).

To date, no consensus exists for managing CFFT. While anticoagulation treatment may help some patients, surgery and endovascular therapy have been found to be beneficial in stroke prevention, underscoring the need for personalized approaches (Muller et al., 2022). This report details a rare presentation of embolic TL, characterized by both a mobile carotid thrombus and distal LVO, requiring careful diagnostic evaluation and a multi-faceted endovascular treatment plan. The thrombotic burden in this patient was distributed across multiple sites, including a mural thrombus at the brachiocephalic bifurcation involving the origins of the right common carotid and right subclavian arteries, a free-floating thrombus at the carotid bifurcation, and an additional thrombus occluding the right ICA terminus and the origin of the M1 segment. This complex anatomical distribution and morphologic diversity of thrombi posed substantial challenges for pre-intervention planning and intraprocedural strategy. Ultimately, successful recanalization was achieved using a hybrid endovascular approach, highlighting the value of case-specific evaluation and procedural adaptability in complex stroke syndromes.

## 2 Case presentation

### 2.1 Clinical presentation and baseline imaging

A 73-year-old man with a 19-year history of atrial fibrillation (dabigatran 110 mg once daily) and well-controlled hypertension (amlodipine 5 mg daily) was admitted to the cardiology service with palpitations and discomfort. He awoke the next morning with new left-sided weakness (last known well 23:00). At 07:30, neurological examination showed left hemiplegia (MRC grade 0/5 in the upper and lower limbs), decreased superficial sensation on the left side, and mild dysarthria. NIHSS was 10 (arm 4, leg 4, dysarthria 1, sensory 1).

**Figure 1:**
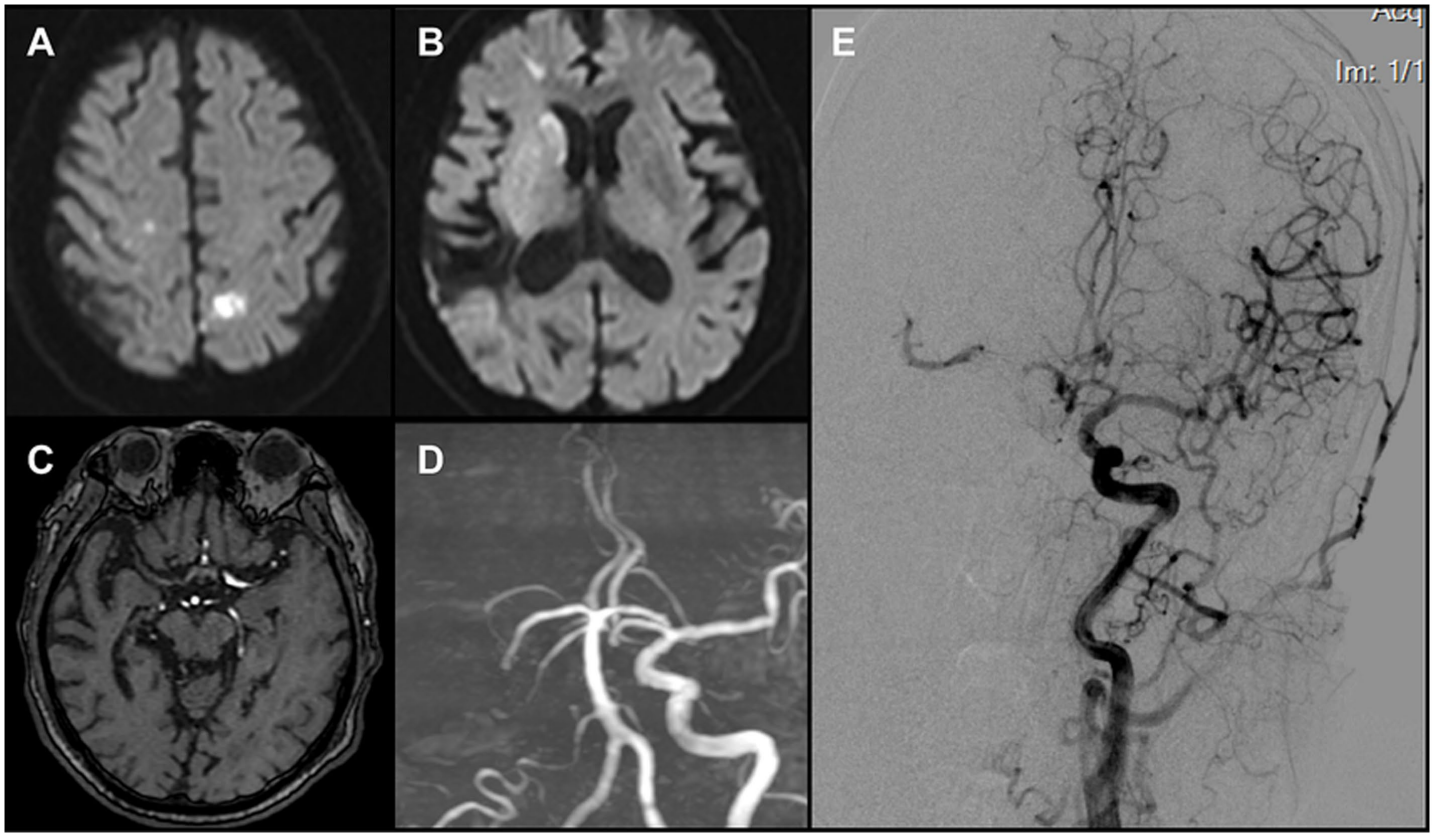
**(A,B)** DWI revealed multiple acute infarcts involving the right caudate head and the bilateral frontal and parietal cortices. **(C)** Original MRA images demonstrated non-visualization of the right middle cerebral artery (MCA). **(D)** TOF 3D reconstruction demonstrated non-visualization of the right internal carotid artery (ICA) and the right MCA **(E)** Left carotid angiography indicated a patent anterior communicating artery. A thrombus was identified in the proximal right MCA, causing subtotal occlusion, while compensatory flow to the MCA territory was provided by the collateral circulation.

Emergency MRI at 07:50 demonstrated multiple acute infarcts in the right caudate head and bilateral frontal and parietal cortices (Figures 1A,B). MRA did not visualize the right ICA or MCA (Figures 1C,D). A diagnosis of acute large-vessel occlusion ischemic stroke was made. Given the patient’s long-standing atrial fibrillation, the TOAST classification suggested a cardioembolic etiology. In this context, the occlusion was considered most likely at the ICA terminus. Because of the mismatch between infarct volume and clinical deficit, the case fulfilled DAWN trial criteria for mechanical thrombectomy. Consent for urgent endovascular intervention was obtained from the patient’s family.

### 2.2 Procedure initiation and FFT identification

The patient arrived in the angiography suite at 08:30, and an 8F sheath was introduced via the right femoral artery. Using a multipurpose diagnostic catheter, a Neuron MAX 088 guiding catheter (Penumbra, USA) was advanced into the left CCA. Angiography demonstrated a patent left ICA with cross-flow via the anterior communicating artery to the right hemisphere. A large thrombus was visualized in the proximal right MCA, causing near-complete occlusion and sluggish forward flow (ASITN/SIR grade 3) (Figure 1E).

**Figure 2:**
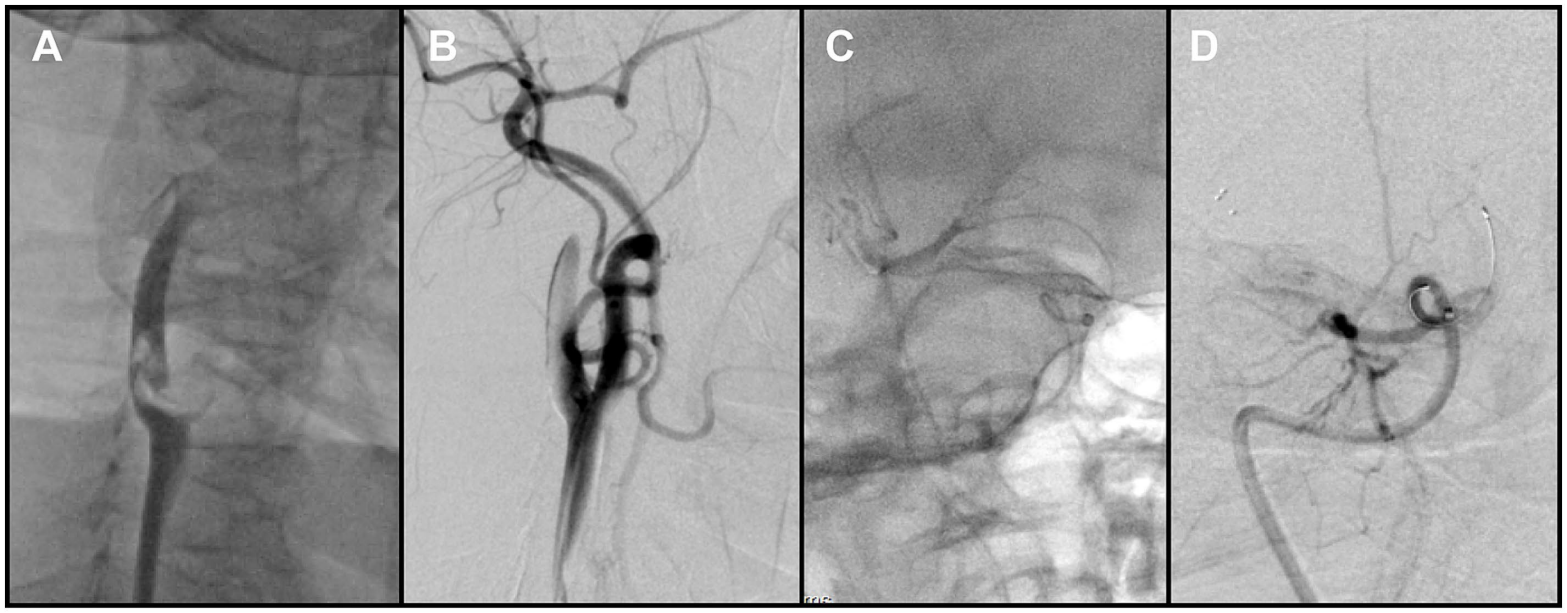
**(A)** Right carotid angiography revealed a free-floating thrombus at the carotid bifurcation. **(B)** Following removal of the proximal thrombus, angiography showed persistent non-opacification of the distal right internal carotid artery (ICA). **(C)** Contrast injection after crossing of the occluded segment by the microcatheter delineated the distal vessel. **(D)** Post-stent deployment angiography demonstrated absence of antegrade flow across the occluded segment.

The Neuron MAX catheter was then advanced into the distal right CCA near the bifurcation. Before performing a complete diagnostic angiogram, a contrast injection was used to confirm catheter position. At this point, a large FFT was identified at the carotid bifurcation, involving into the origins of both the ICA and the ECA. The thrombus exhibited pulsatile motion synchronous with the cardiac cycle, consistent with a mobile/mural thrombus (Figure 2A). At the same time, antegrade flow in the ICA was stagnant, suggesting distal ICA occlusion. The overall presentation was therefore consistent with a tandem embolic lesion in the anterior circulation. Given the presence of collateral circulation, a proximal-to-distal treatment strategy was adopted.

Aspiration thrombectomy was attempted through the Neuron MAX catheter, but resistance was encountered, suggesting catheter-tip or intraluminal clot occlusion. Continuous and pulsed aspiration failed to restore flow, and blind catheter withdrawal was avoided due to the risk of embolic escape. Therefore, a Solitaire 4 × 20 mm stent retriever (Medtronic, USA) was deployed at the tip of the Neuron MAX guiding catheter. With continuous aspiration applied through the guiding catheter, the stent was used to scrape and engage the obstructing thrombus. This maneuver was repeated three times, after which the guiding catheter was successfully cleared and patency restored. Subsequent angiography demonstrated complete removal of the thrombus at the bifurcation of the right internal carotid artery (Figure 2B).

### 2.3 Intracranial flow restoration and proximal lesion

After restoration of catheter patency, the Neuron MAX catheter was navigated into the ICA. A Catalyst 6 aspiration catheter (Stryker Neurovascular, USA) was advanced and used to aspirate thrombotic material from segments C1 through C4 of the right ICA, with adequate backflow but no significant thrombus was retrieved. The microcatheter and microwire were then used to traverse the terminal ICA occlusion and advance into the distal M1 segment (Figure 2C). Following deployment of the Solitaire stent retriever, angiography still showed no antegrade flow (Figure 2D). Because of the heavy thrombus burden, a combined approach was adopted, retrieving the stent retriever together with en bloc withdrawal of the aspiration catheter.

**Figure 3:**
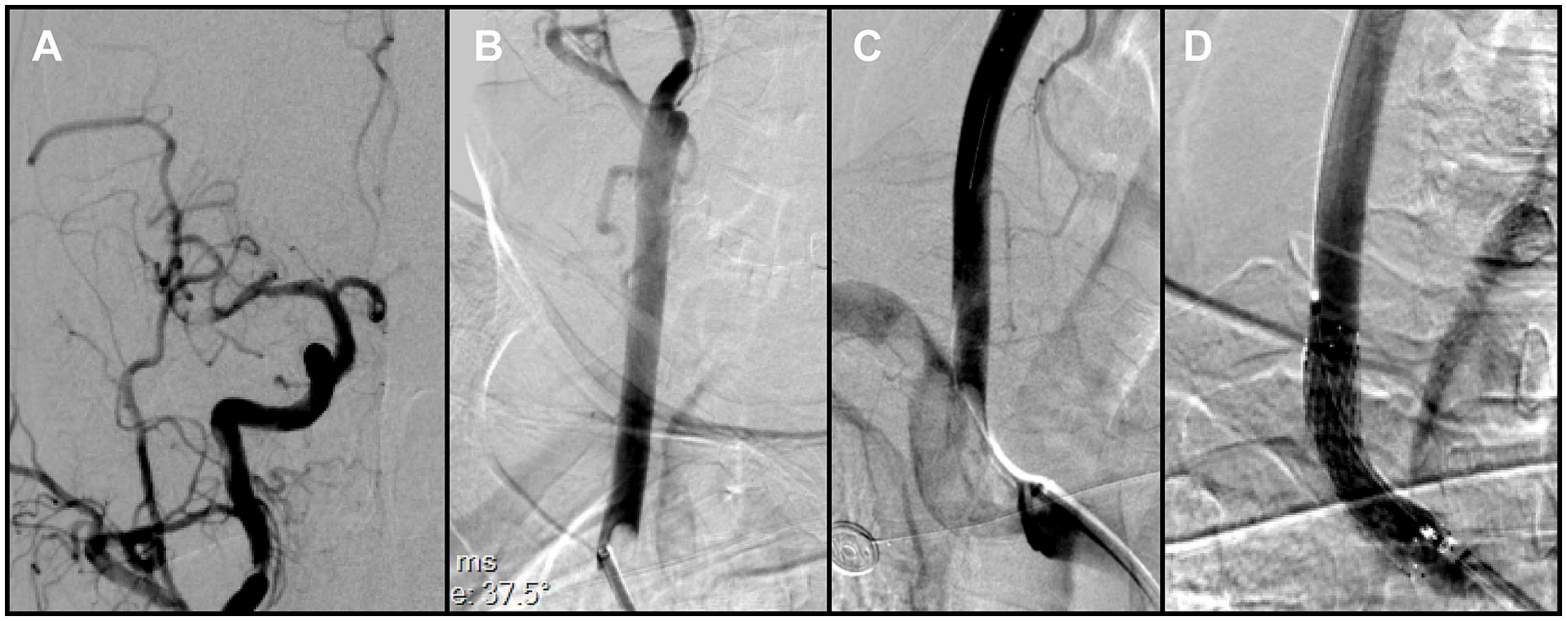
**(A)** Right common carotid angiography showed a small residual thrombus at the distal right middle cerebral artery (MCA) that did not significantly compromise hemodynamics. **(B)** A large thrombus was identified in the right common carotid artery, adjacent to the delivery catheter. **(C)** Brachiocephalic angiography with distal protection demonstrated a large free-floating thrombus (FFT) involving the brachiocephalic bifurcation, extending into the origins of the right common carotid and right subclavian arteries. **(D)** Post-stenting angiography demonstrated full expansion of the stent in the right common carotid artery (CCA), without residual stenosis at the treatment site.

During this phase, impaired backflow in the Neuron MAX catheter was observed. After the catheter was withdrawn to the common carotid artery, adequate backflow through the guiding catheter was restored. Catheter angiography demonstrated severe vasospasm of the right ICA, explaining the earlier loss of backflow. In addition, a small residual thrombus was detected in the distal M1 segment (Figure 3A), and a previously unrecognized thrombus was identified in the right CCA (Figure 3B). After discussion, the team decided to prioritize treatment of the intracranial lesion. However, when the catheter was advanced into the ICA for follow-up angiography, the residual thrombus was found to have migrated spontaneously into an M4 cortical branch, and the ICA vasospasm had also resolved spontaneously. At that point, intracranial reperfusion was satisfactory, with an mTICI score of 2b, and no further treatment was required.

### 2.4 Management of proximal FFT

To reduce the risk of embolization during assessment of the proximal vessels, a Solitaire stent retriever was temporarily deployed in the ICA to provide distal protection. As the Neuron MAX catheter was withdrawn to the brachiocephalic artery for angiography, a large FFT, corresponding to the lesion previously noted in the right CCA but only partially visualized, was clearly delineated at the brachiocephalic bifurcation, involving the origins of both the right subclavian artery and the right common carotid artery (Figure 3C). This thrombus at the brachiocephalic bifurcation was considered pre-existing, since no manipulation of the right subclavian artery had occurred before this angiogram.

**Figure 4:**
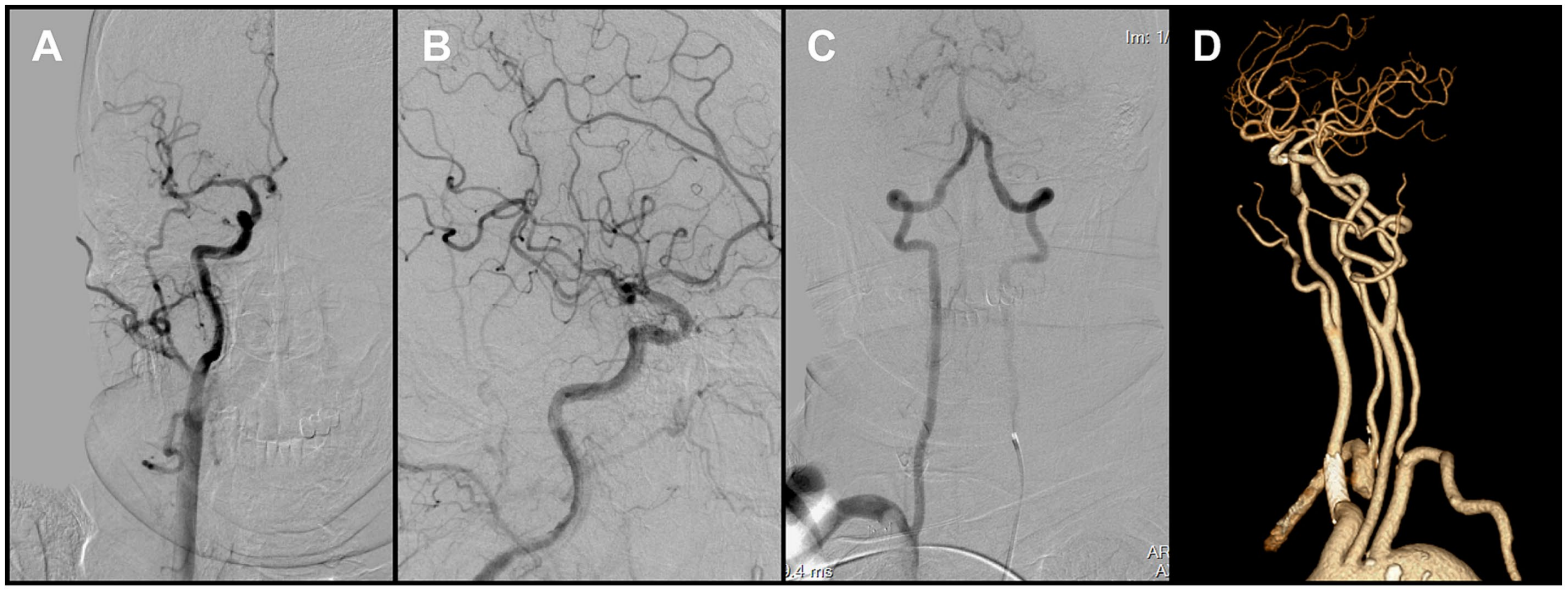
**(A,B)** Final angiography prior to procedural completion demonstrated reperfusion of the right carotid system (mTICI grade 2C). **(C)** Left vertebral angiography showed retrograde flow in the right vertebral artery. **(D)** Follow-up CTA at 1 month demonstrated persistent occlusion of the right subclavian artery.

Because en bloc retrieval across the bifurcation carried high risk of embolic escape and recurrent cerebral embolism, an *in situ* stabilization strategy was selected. Under distal protection with a SpiderFX device, a self-expanding PROTÉGÉ 9 × 40 mm stent (Medtronic, USA) was deployed in the CCA to pin and compress the thrombus against the vessel wall without predilation. Post-deployment angiography confirmed the ICA remained widely patent, with no residual stenosis (Figure 3D). The previously noted intracranial emboli had resolved spontaneously, and the final reperfusion grade improved to mTICI 2C (Figures 4A,B). Before completion of the procedure, angiography of the left vertebral artery demonstrated patent posterior circulation vessels with retrograde opacification of the right vertebral artery, indicating preserved collateral flow and minimal risk of thrombus migration from the right subclavian artery into the intracranial circulation (Figure 4C).

### 2.5 Intraoperative step-by-step sequence

1. Via a right femoral approach, a multipurpose diagnostic catheter was used to advance a Neuron MAX 088 guiding catheter to the left common carotid artery (CCA) for angiography, which demonstrated collateral cross-flow.
2. The catheter was then repositioned into the right CCA, where a free-floating thrombus (FFT) at the carotid bifurcation was identified.
3. Aspiration through the Neuron MAX failed due to catheter-tip occlusion; a Solitaire 4 × 20 mm stent retriever was deployed to clear the catheter and restore patency.
4. A Catalyst 6 aspiration catheter was advanced for internal carotid artery (ICA) aspiration. Combined stent-retriever–assisted aspiration targeted thrombus at the ICA terminus and proximal M1.
5. During this phase, loss of backflow occurred in the Neuron MAX; after withdrawal to the CCA, angiography showed a small residual M1 thrombus, severe ICA vasospasm, and a large thrombus in the right CCA.
6. After brief observation, ICA vasospasm resolved and the residual M1 thrombus migrated distally, obviating further intracranial thrombectomy (intracranial reperfusion mTICI 2b at that time).
7. A Solitaire stent retriever was temporarily deployed in the ICA at the C1 segment for distal protection, while the Neuron MAX catheter was withdrawn to the brachiocephalic artery to perform angiography, which fully delineated the bifurcation thrombus.
8. Under additional distal protection with a SpiderFX device placed in the cervical ICA, a PROTÉGÉ 9 × 40 mm self-expanding stent was deployed in the right CCA without predilation to pin the brachiocephalic-bifurcation FFT against the vessel wall.
9. Post-stenting angiography confirmed full stent expansion and a widely patent ICA with no residual stenosis; overall final reperfusion was graded mTICI 2C.

### 2.6 Antithrombotic therapy and outcomes

Following stent deployment, tirofiban was given as a loading infusion (0.4 μg/kg/min for 30 min) followed by continuous infusion (0.1 μg/kg/min). To reduce the risk of further cardioembolic events, therapeutic low-molecular-weight heparin (100 IU/kg once daily) was added postoperatively. During the first 24 h, platelet function and coagulation parameters were monitored every 12 h. At 24 h, follow-up non-contrast CT revealed a localized PH1-type hemorrhage as defined by ECASS. In accordance with our institutional protocol, tirofiban was discontinued and replaced with oral clopidogrel 75 mg once daily, while low-molecular-weight heparin was continued. On postoperative day 3, repeat CT showed partial resolution of the hemorrhage, indicating reduced periprocedural bleeding risk. The regimen was therefore adjusted to clopidogrel 75 mg once daily in combination with dabigatran 110 mg twice daily. According to institutional practice, this dual therapy was planned for 3–6 months, with follow-up CTA at 1 month and 6 months, and DSA at 12 months to confirm stent apposition and thrombus resolution.

Follow-up CTA at 1 month confirmed persistent occlusion of the right subclavian artery (Figure 4D). Clinically, the patient remained asymptomatic, with no upper-limb ischemic symptoms and no significant inter-arm blood pressure difference. Conservative management was therefore adopted, with surveillance imaging as planned. Referral for vascular surgical or endovascular revascularization was reserved for the development of arm ischemia, disabling symptoms, or a hemodynamically significant inter-arm pressure discrepancy. The patient showed marked neurological improvement and was discharged on the above regimen. At the 3-month clinical follow-up, the modified Rankin Scale (mRS) score was 0, indicating complete functional recovery.

## 3 Discussion and conclusion

This case highlights a rare presentation of cardioembolic free-floating thrombus (FFT) characterized by sequential involvement of multiple bifurcation sites. Unlike most reported FFTs that are confined to a single carotid segment, our patient exhibited tandem occlusions affecting the brachiocephalic bifurcation, the carotid bifurcation, and the terminal ICA bifurcation. This uncommon multi-segment pattern created significant diagnostic uncertainty and posed substantial technical challenges during endovascular management.

In acute stroke intervention, comprehensive vascular imaging from the aortic arch to the intracranial circulation is essential. In this case, preoperative evaluation was limited to cranial MRI and intracranial MRA, without detailed assessment of the cervical vessels or collateral pathways. Consequently, the heavy thrombus burden in the proximal arteries was not identified before the procedure, which increased both technical complexity and procedural risk. This case emphasizes that when initial noninvasive imaging is incomplete or inconclusive, early intraoperative angiography should be performed to clarify vascular anatomy, guide catheter selection, and anticipate thrombus burden (Asano et al., 2021).

We speculate that this case represented a cardioembolic FFT affecting the entire right carotid system, with thrombi lodged at three successive bifurcation sites—the brachiocephalic bifurcation into the subclavian and common carotid arteries, the carotid bifurcation into the internal and external carotid arteries, and the terminal bifurcation of the internal carotid artery. We hypothesize that a single large cardioembolic FFT became intermittently trapped at these branching points, fragmented, and subsequently migrated distally, resulting in the sequential involvement of multiple bifurcation sites observed in this patient. The proximal thrombus near the brachiocephalic bifurcation was recognized only late during the procedure because preoperative imaging did not include evaluation of the aortic arch and supra-aortic trunks. However, as the procedure did not involve manipulation of the right subclavian artery, the lesion was unlikely to represent a newly formed iatrogenic clot. Importantly, the thrombi at both the brachiocephalic and carotid bifurcations appeared as free-floating thrombi rather than occlusive lesions, consistent with the characteristics of cardioembolic FFTs described in prior reports (Bhatti et al., 2007; Fridman et al., 2019). Cardioembolic FFTs are generally fresh, friable, and loosely adherent, in contrast to the dense and persistent thrombi arising from atherosclerotic plaque rupture (Jayyusi et al., 2024). Their fragile and mobile nature provides a plausible explanation for how a single cardioembolic clot could progressively fragment and become sequentially trapped at multiple branching sites from the aortic arch to the intracranial vasculature (Carr et al., 2018; Yokoyama et al., 2023). Furthermore, the absence of residual stenosis after direct stent deployment at the carotid bifurcation without predilation further supports the non-atherosclerotic nature of the thrombus.

The optimal management of FFT remains controversial, as no randomized trials or unified guidelines are currently available. Observational studies suggest that both anticoagulation (ACT) and antiplatelet therapy (APT) can promote thrombus regression with comparable efficacy in many cases (Bhatti et al., 2007; Fridman et al., 2019). More recent systematic reviews indicate that a combined regimen of ACT, APT, and statins is associated with higher rates of thrombus resolution and lower recurrence risk (Jayyusi et al., 2024). Given the particularly high risk of recurrence within the first week after diagnosis, an anticoagulation-based strategy appears to be a prudent initial approach to stabilize the lesion (Muller et al., 2022). The role of surgical or endovascular revascularization is more complex: while urgent carotid endarterectomy or stenting may be warranted in patients with severe residual stenosis, early intervention carries an inherent risk of thrombus dislodgement and cerebral embolization (Bhogal et al., 2021). Accordingly, a stepwise strategy—initial intensive antithrombotic therapy to reduce clot burden, followed by delayed revascularization if significant stenosis persists—has been advocated as a pragmatic approach in reviews and case series.

In practice, however, urgent endovascular treatment may be required in cases of disabling stroke, as in our patient, and several procedural strategies have been described for FFT. Large-bore aspiration catheters can be effective in selected cases but often fail with heavy clot burden, necessitating adjuncts such as proximal balloon occlusion with flow reversal or distal filter protection to mitigate the risk of embolic migration (Carr et al., 2018). Stent retrievers used in conjunction with protection devices have also achieved favorable outcomes (Giragani et al., 2017; Yamamoto et al., 2023). For large or bifurcation thrombi, dual-layer stent retrievers such as the EmboTrap III, especially when combined with balloon-guided catheters (BGCs), have enabled complete removal without distal embolization (Yokoyama et al., 2023). Ultrasound-assisted aspiration has been proposed as a novel adjunct to optimize catheter alignment in anatomically challenging cases (Wang et al., 2022). In addition, carotid stenting to trap the thrombus against the vessel wall has been reported as a definitive treatment in small case series (Bhogal et al., 2021).

Although various endovascular approaches have been reported with favorable outcomes, our case posed additional challenges, as thrombi were encountered at three separate bifurcation sites. We prioritized rapid recanalization of the intracranial circulation by clearing thrombi at the ICA terminus and MCA, which was justified in the emergency setting. However, the absence of balloon-guided catheter protection likely increased the risk of intra-procedural embolization. In this case, a BGC was not used because the device was not available at our institution at the time of the procedure. For the thrombus at the brachiocephalic bifurcation, we ultimately deployed a self-expanding stent in the CCA to compress the clot against the vessel wall, thereby preventing potential embolic migration. The decision to proceed with immediate stenting was made intraoperatively for several reasons. First, the thrombus at the brachiocephalic bifurcation was large and involved both the right subclavian and common carotid arteries, making safe and complete extraction technically unfeasible. Second, although a medical-first approach with anticoagulation was considered since antegrade flow was not severely impaired, the risk of recurrent embolization into the intracranial circulation was judged to be unacceptably high. Finally, while previous reports have described successful thrombus resolution with anticoagulation in cardioembolic FFT (Jayyusi et al., 2024), those cases typically involved patients with mild or stable symptoms, in contrast to our patient with a disabling presentation. For these reasons, acute stent deployment was selected to secure the lesion and reduce immediate embolic risk. Taken together, our experience underscores that FFT management cannot be guided by a single algorithm but instead requires individualized procedural planning, thorough preprocedural assessment, and flexible adaptation of available endovascular techniques.

Postprocedural antithrombotic management in this case was particularly complex due to the dual need for antiplatelet therapy following carotid artery stenting and anticoagulation for underlying atrial fibrillation. While traditional triple therapy (oral anticoagulant plus dual antiplatelet therapy) offers comprehensive thromboembolic protection, it carries a significantly increased bleeding risk. A retrospective study by Pardo-Galiana et al. (2022) demonstrated that dual therapy combining a DOAC with clopidogrel significantly reduced bleeding complications compared to triple therapy (1-month bleeding rate: 0% vs. 23.8%), without increasing the incidence of recurrent embolism. Based on this evidence, the patient was prescribed a secondary prevention regimen of clopidogrel 75 mg once daily plus dabigatran 110 mg twice daily after the procedure. Notably, the patient had been treated for cerebral embolism at a different hospital 6 months earlier, where he was prescribed a secondary prevention regimen of dabigatran 110 mg once daily. Follow-up assessments indicated that this reduced dose had failed to prevent recurrence of the cerebral embolism. Previous studies have suggested that unindicated dose reduction or escalation of DOACs is not associated with increased safety and may, in fact, increase the risk of adverse events (Zhang et al., 2021). Therefore, in this case we chose to resume the standard dose (110 mg twice daily) in combination with antiplatelet therapy to balance the risks of bleeding and recurrence. At the 3-month follow-up, the patient had experienced no ischemic or hemorrhagic events, suggesting that this secondary prevention regimen was both effective and safe in this case.

In conclusion, this case highlights a rare presentation of cardioembolic FFT with sequential involvement of multiple bifurcation sites. It underscores the importance of comprehensive vascular imaging, individualized endovascular strategies, and carefully tailored antithrombotic therapy. Long-term surveillance of the untreated subclavian occlusion, integrating both clinical and imaging follow-up, was also planned. These insights may help refine diagnostic and therapeutic approaches in similarly complex presentations.

## Data Availability

The raw data supporting the conclusions of this article will be made available by the authors, without undue reservation.

## References

[ref1] Asano H. Shimizu T. Aihara M. Yamaguchi R. Aishima K. Yoshimoto Y. (2021). Acute endovascular revascularization for patients with common carotid artery occlusion apparent on cervical magnetic resonance angiography. J. Stroke Cerebrovasc. Dis. 30:105626. doi: 10.1016/j.jstrokecerebrovasdis.2021.105626, PMID: 33516069

[ref2] Assis Z. Menon B. K. Goyal M. Demchuk A. M. Shankar J. Rempel J. L.. (2018). Acute ischemic stroke with tandem lesions: technical endovascular management and clinical outcomes from the ESCAPE trial. J. Neurointerv. Surg. 10, 429–433. doi: 10.1136/neurintsurg-2017-013316, PMID: 29021311

[ref3] Bhatti A. F. Leon L. R. Jr. Labropoulos N. Rubinas T. L. Rodriguez H. Kalman P. G.. (2007). Free-floating thrombus of the carotid artery: literature review and case reports. J. Vasc. Surg. 45, 199–205. doi: 10.1016/j.jvs.2006.09.057, PMID: 17210411

[ref4] Bhogal P. AlMatter M. Aguilar Perez M. Bazner H. Henkes H. Hellstern V. (2021). Carotid stenting as definitive treatment for free floating thrombus-review of 7 cases. Clin. Neuroradiol. 31, 449–455. doi: 10.1007/s00062-020-00898-y, PMID: 32221623 PMC8211580

[ref5] Carr K. Tew D. Becerra L. Siddall K. Dubensky L. Serulle Y. (2018). Endovascular aspiration of a symptomatic free-floating common carotid artery thrombus. Neuroradiology 60, 1103–1107. doi: 10.1007/s00234-018-2077-2, PMID: 30109383

[ref6] Dowlatshahi D. Lum C. Menon B. K. Bharatha A. Dave P. Puac-Polanco P.. (2023). Aetiology of extracranial carotid free-floating thrombus in a prospective multicentre cohort. Stroke Vasc. Neurol. 8, 194–196. doi: 10.1136/svn-2022-001639, PMID: 36368714 PMC10359789

[ref7] Fridman S. Lownie S. P. Mandzia J. (2019). Diagnosis and management of carotid free-floating thrombus: a systematic literature review. Int. J. Stroke 14, 247–256. doi: 10.1177/1747493019828554, PMID: 30722756

[ref8] Giragani S. Balani A. Agrawal V. (2017). Stentriever thrombectomy with distal protection device for carotid free-floating thrombus: a technical case report. J. Neurointerv. Surg. 9:e25. doi: 10.1136/neurintsurg-2016-012904.rep, PMID: 28122915

[ref9] Jayyusi F. AlBarakat M. M. Al-Rousan H. H. Alawajneh M. M. Alkasabrah A. R. Abujaber M.. (2024). The efficacy of medical interventions for free-floating thrombus in cerebrovascular events: a systematic review. Brain Sci. 14:801. doi: 10.3390/brainsci14080801, PMID: 39199493 PMC11352359

[ref10] Muller M. D. Raptis N. Mordasini P. Z'Graggen W. Raabe A. Schucht P.. (2022). Natural history of carotid artery free-floating thrombus-a single center, consecutive cohort analysis. Front. Neurol. 13:993559. doi: 10.3389/fneur.2022.993559, PMID: 36237628 PMC9553207

[ref11] Papanagiotou P. Haussen D. C. Turjman F. Labreuche J. Piotin M. Kastrup A.. (2018). Carotid stenting with antithrombotic agents and intracranial thrombectomy leads to the highest recanalization rate in patients with acute stroke with tandem lesions. JACC Cardiovasc. Interv. 11, 1290–1299. doi: 10.1016/j.jcin.2018.05.036, PMID: 29976365

[ref12] Pardo-Galiana B. Medina-Rodriguez M. Millan-Vazquez M. Cabezas-Rodriguez J. A. Lebrato-Hernandez L. Ainz-Gomez L.. (2022). Antithrombotic treatment after carotid stenting in patients with concomitant atrial fibrillation. AJNR Am. J. Neuroradiol. 43, 727–730. doi: 10.3174/ajnr.A7482, PMID: 35393364 PMC9089259

[ref13] Sharma R. Lee K. (2025). Advances in treatments for acute ischemic stroke. BMJ 389:e076161. doi: 10.1136/bmj-2023-076161, PMID: 40335091

[ref14] Wang P. Wang Z. Pan J. Lu K. Sun L. Geng Y. (2022). Case report: ultrasound-assisted endovascular therapy for carotid artery floating thrombus. Front. Cardiovasc. Med. 9:961760. doi: 10.3389/fcvm.2022.961760, PMID: 36187000 PMC9519131

[ref15] Yamamoto Y. Yamamoto N. Matsuda T. Kuroda K. Yamaguchi I. Sogabe S.. (2023). Stent retrieval for free-floating thrombus attached to carotid artery stenosis: a report of two cases. Surg. Neurol. Int. 14:274. doi: 10.25259/SNI_513_2023, PMID: 37680937 PMC10481818

[ref16] Yokoyama R. Haraguchi K. Ogane K. Imataka S. Nakamura Y. Hanyu N.. (2023). Mechanical thrombectomy using a large dual-layer stent retriever for near-occlusion of the common carotid bifurcation caused by a giant free-floating thrombus. J. Neuroendovasc. Ther. 17, 293–298. doi: 10.5797/jnet.cr.2023-0050, PMID: 38125958 PMC10730296

[ref17] Zhang X. L. Zhang X. W. Wang T. Y. Wang H. W. Chen Z. Xu B.. (2021). Off-label under- and overdosing of direct oral anticoagulants in patients with atrial fibrillation: a meta-analysis. Circ. Cardiovasc. Qual. Outcomes 14:e007971. doi: 10.1161/CIRCOUTCOMES.121.007971, PMID: 34932377

