# Correction: Endovascular management of tandem embolic stroke due to cardioembolic free-floating thrombus: a case report

**DOI:** 10.3389/fnins.2025.1729599

**Published:** 2025-10-31

**Authors:** Frontiers Production Office

**Affiliations:** Frontiers Media SA, Lausanne, Switzerland

**Keywords:** embolic tandem lesion, free-floating thrombus, mechanical thrombectomy, stroke, case report

For the reviewer Yuzhou Wang, the affiliation The Sixth Affiliated Hospital, Sun Yat-sen University, China was erroneously given as School of Business Administration, China.

The original version of this article has been updated.

