# Supplementary figures and images for: Endovascular management of tandem embolic stroke due to cardioembolic free-floating thrombus: a case report
Source: Front Neurosci. 2025 Oct 15;19:1654601. doi: 10.3389/fnins.2025.1654601 (PMC12568574; doi:10.3389/fnins.2025.1654601)

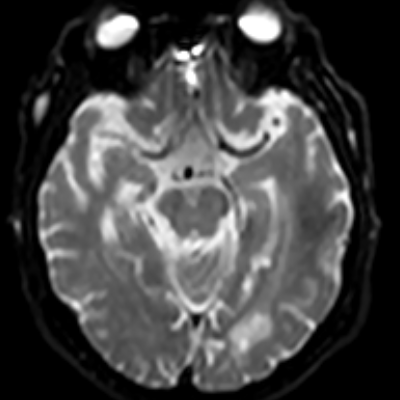

Supplement: Supplementary file 1 [file Image_1.PNG]
